# Financial Toxicity and Out-of-Pocket Costs for Patients with Head and Neck Cancer

**DOI:** 10.3390/curroncol30050371

**Published:** 2023-05-10

**Authors:** Justin Smith, Justin Yu, Louisa G. Gordon, Madhavi Chilkuri

**Affiliations:** 1 Townsville University Hospital, Townsville, QLD 4814, Australia; 2 College of Medicine and Dentistry, James Cook University, Townsville, QLD 4811, Australia; 3 Health Economics, Population Health Program, QIMR Berghofer Medical Research Institute, Brisbane, QLD 4006, Australia; 4 School of Public Health, The University of Queensland, Brisbane, QLD 4006, Australia; 5 School of Nursing, Queensland University of Technology, Brisbane, QLD 4059, Australia

**Keywords:** head and neck cancer, financial toxicity, out-of-pocket expenses, survivorship, quality of life

## Abstract

Aim: To quantify financial toxicity and out-of-pocket costs for patients with HNC in Australia and explore their relationship with health-related quality of life (HRQoL). Methods: A cross-sectional survey was administered to patients with HNC 1–3 years after radiotherapy at a regional hospital in Australia. The survey included questions on sociodemographics, out-of-pocket expenses, HRQoL, and the Financial Index of Toxicity (FIT) tool. The relationship between high financial toxicity scores (top quartile) and HRQoL was explored. Results: Of the 57 participants included in the study, 41 (72%) reported out-of-pocket expenses at a median of AUD 1796 (IQR AUD 2700) and a maximum of AUD 25,050. The median FIT score was 13.9 (IQR 19.5) and patients with high financial toxicity (*n* = 14) reported poorer HRQoL (76.5 vs. 114.5, *p* < 0.001). Patients who were not married had higher FIT scores (23.1 vs. 11.1, *p* = 0.01), as did those with lower education (19.3 vs. 11.1, *p* = 0.06). Participants with private health insurance had lower financial toxicity scores (8.3 vs. 17.6, *p* = 0.01). Medications (41%, median AUD 400), dietary supplements (41%, median AUD 600), travel (36%, median AUD 525), and dental (29%, AUD 388) were the most common out-of-pocket expenses. Participants living in rural locations (≥100 km from the hospital) had higher out-of-pocket expenses (AUD 2655 vs. AUD 730, *p* = 0.01). Conclusion: Financial toxicity is associated with poorer HRQoL for many patients with HNC following treatment. Further research is needed to investigate interventions aimed at reducing financial toxicity and how these can best be incorporated into routine clinical care.

## 1. Introduction

Head and neck cancer (HNC) is a heterogenous group of tumours that arise from the oral cavity, larynx, nasopharynx, oropharynx, or hypopharynx [1]. Treatment often consists of a multi-modal approach with various combinations of surgery, radiotherapy, and chemotherapy [2]. As a result of treatment and its associated side effects, patients with HNC often require intensive multi-disciplinary care both during and after treatment, which results in significant healthcare utilisation and cost to both the healthcare system and patients [3]. During radiotherapy, patients often experience acute side effects such as mucositis, dysphagia, or odynophagia which generally requires analgesic medications and dietary supplements. Long-term survivorship care of patients with HNC is complex and involves a multi-disciplinary approach. For example, patients who have received radiotherapy require regular dental follow-up given the increased risk of periodontal disease, dental caries, and osteoradionecrosis [4]. 

It is increasingly recognised that a significant number of cancer survivors experience ‘financial toxicity’ as a result of their cancer diagnosis and treatment, which can cause a reduction in quality of life (QoL) and clinical outcomes [5]. Financial toxicity is defined as the hardship or distress that arises from the financial burden of cancer treatment [6]. This arises from a combination of high out-of-pocket expenses associated with treatment and also a reduction in income through taking time off work. Patients with financial toxicity have been demonstrated to delay their treatment, not fill medication scripts, rationalise medications, or forgo treatment altogether, all of which contribute to poorer clinical outcomes [6]. The financial burden for patients with HNC is particularly relevant given that this malignancy has high healthcare needs associated with treatment and can result in long-term functional deficits, such as those impacting upon voice, cosmesis, or swallowing [7]. A study in patients with HNC demonstrated that financial toxicity was associated with reduced overall survival and cancer-specific survival [8]. 

The majority of studies associated with financial toxicity for patients with HNCs have been conducted in the USA [9,10,11,12]. There has also been research performed in Canada, with a study by Hueniken et al. developing the Financial Index of Toxicity (FIT) instrument for use in patients with HNC [13]. There have been few studies investigating financial toxicity for patients with HNC in publicly funded healthcare systems such as Australia. Although Australia has a publicly funded healthcare system, items such as travel, accommodation, and dental care still incur significant costs for patients, as well as the need to take time off work during treatment and recovery. Additionally, the financial burden on patients with HNC from regional and rural areas remains under-studied, with these patients likely to be disproportionately affected. Defining the prevalence of financial toxicity and out-of-pocket costs for patients with HNC will allow for the identification of areas that require further intervention to reduce financial burdens. 

The aim of this study was to quantify and describe the financial toxicity and out-of-pocket costs for patients with HNC in regional Australia and to consider their relationship with quality of life.

## 2. Materials and Methods

### 2.1. Design and Setting

This study was a cross-sectional study conducted at the Townsville University Hospital (TUH) in Queensland, Australia. TUH is a regional tertiary hospital that treats patients with HNC from a wide catchment area in Northern Queensland. This is a public hospital that is co-funded by the Australian and state governments. Patients with private health insurance are also treated at this facility (covering in-patient services only) and there are no other public or private radiation oncology services in Townsville. Ethics approval was granted by Townsville Hospital and Health Service Human Research Ethics Committee (HREC/QTHS/76534). The Strengthening the Reporting of Observational studies in Epidemiology (STROBE) guidelines for reporting observational studies was utilized [14].

### 2.2. Overview of Australian Healthcare System

Australia has a universal healthcare system called Medicare which is funded by Commonwealth and state/territory governments through income tax revenue. The Medicare system provides coverage for treatment provided in public hospitals, subsidies for healthcare services provided out of hospitals (e.g., general practitioner appointments), and a discount on prescription medicines. Australia also has a private medical system in which medical professionals operate in a fee-for-service environment. Patients are required to pay an out-of-pocket expense if the doctor’s fee is higher than the Medicare rebate. Medical practitioners can opt to accept the Medicare rebate as the fee, which results in no out-of-pocket expense (referred to as bulk-billing). Currently, Medicare has limited coverage for services such as dental care [15]. Australians can elect to purchase private health insurance, which allows additional coverage for services such as dental and physiotherapy, and private hospitals [16].

### 2.3. Participants and Recruitment

Patients with head and neck cancer who completed treatment at the TUH were considered eligible for inclusion if they met the following criteria:

- Completed radiotherapy for mucosal SCC of the head and neck region (either as definitive or adjuvant treatment ± chemotherapy);
- One to three years after completion of radiotherapy treatment;
- Aged >18 years;
- Disease-free and well-enough to complete the survey as determined by the study investigator.

All patients who had completed radiotherapy one to three years previously at TUH (November 2018 to November 2020) were identified through review of the hospital oncology management information system. This electronic medical records system captures all patients treated with radiotherapy at TUH. Patients deemed eligible were then contacted by a study investigator by phone to discuss the study requirements. Informed consent for participation was obtained verbally (for patients completing the survey over the phone or electronically) or through signing of a consent form for those completing in person or by postal mail.

### 2.4. Data Collection

Patients who agreed to participate completed a questionnaire either over the phone, electronically (through email or RedCap), via postal mail, or in-person at a clinic appointment. The survey given to the patients (Supplementary Questionnaire S1) included details about their sociodemographic characteristics (level of education, marital status, smoking status, private health insurance, annual income, employment status, occupation, employment during and after treatment), as well as whether they were asked by their treatment team about any financial concerns and how comfortable they were discussing financial difficulties with their treating medical team. The survey included a section on out-of-pocket expenses within the first 12 months of diagnosis (dental, allied health, mental health, general practitioner, specialist appointments, accommodation, travel, home services, dietary supplements, investigations, medications, and other). Quality of life was assessed using the Functional Assessment of Cancer Therapy—Head and Neck (FACT-H&N) [17]. This questionnaire assesses quality of life across five domains of wellbeing—physical, social/family, emotional, functional, and the head and neck cancer subscale. Each question is answered on a 5-point Likert scale with a possible range of scores from 0 to 156 (higher score indicates a better quality of life). Financial toxicity was evaluated using the Financial Index of Toxicity (FIT) survey, which scores financial toxicity on a scale of 0–100 (higher score indicating higher financial toxicity). This instrument was designed to assess three domains: financial strain (psychological distress associated with finances), financial stress (objective financial burden—which results from out-of-pocket spending on healthcare [18]), and lost productivity [13].

A chart review of electronic medical records was conducted to extract demographical and tumour information such as: distance from town of residence to TUH, Eastern Cooperative Oncology Group (ECOG) status, site of primary tumour, stage (AJCC 8th edition), and date of treatment completion. Data was stored in a RedCap database and exported for analysis.

### 2.5. Statistical Analyses

Descriptive statistics were used to describe the sociodemographic and clinical profiles of the participants. The statistical program Stata V16 was used for analysis. Data were reported as mean and standard deviations for normally distributed continuous variables and median and interquartile range for non-normally distributed continuous variables. Categorical variables were reported as counts and proportions. FIT scores were calculated using instructions provided by the authors of the instrument. Scores were reported as a total score, financial stress score, and lost productivity score. These were graphically represented using box plots. Subgroup analyses investigating clinical and demographical factors associated with financial toxicity were conducted. To determine if there was a difference in financial toxicity scores for each of these factors, a Mann–Whitney U test was used. Categorical variables were collapsed into dichotomous responses when there was more than one response. Results for the FACT H&N were reported as median and interquartile range for all five domains as well as the total score. Participants with the highest financial toxicity scores (top 25%) were defined as “high financial toxicity” and the rest “low financial toxicity”. FIT scores between the two groups were then compared and a Mann–Whitney U test was used to determine if there was a statistically significant difference in scores. The Spearman correlation coefficient was reported for the relationship between FIT score and FACT H&N. Out-of-pocket expenses were reported as the number of participants who reported an expense for each item, as well as the median expense for each item and overall (excluding participants who did not have an out-of-pocket expense). Out-of-pocket costs were reported in 2019/20 Australian dollars (AUD). Clinical and demographical factors associated with out-of-pocket expenses were explored and a Mann–Whitney U test used to determine if there was a statistically significant difference in expenses.

## 3. Results

**Figure 1:**
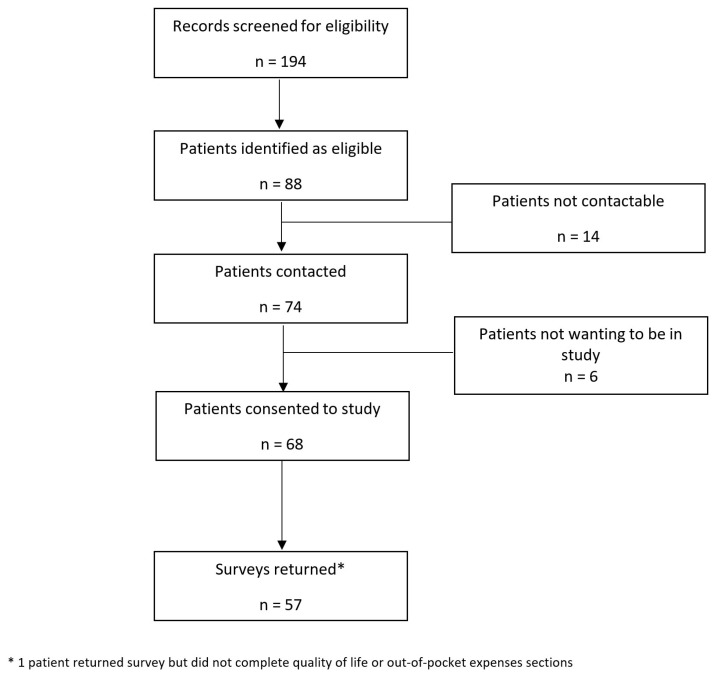
Study Flow Chart.

There were seventy-four patients contacted to discuss the requirements of study, with six patients not willing to participate and eleven patients who did not return the questionnaire (Figure 1). A total of 57 patients (response rate 77%) completed the survey and comprised the cohort for analysis. The mean age of participants at survey completion was 63.8 years and the mean time from completion of treatment to survey was 1.9 years, with a minimum of 1 year and a maximum of 3.1 years (Table 1). Less than half of the participants (46%) had completed tertiary education. Most participants were male (75%), married (58%), and 29 participants were working at time of diagnosis, with most (23/29) employed full-time. Over half of the participants (53%) were receiving a low household annual income (less than AUD 45,000). Only nineteen participants (34%) had private health insurance. The most common head and neck cancer subsite was oropharyngeal (65%). There were 13 people (23%) who reported a reduced income post treatment. Of the twenty-three people working full-time at time of diagnosis, eleven had returned to full-time work by 12 months post treatment (48%), four had returned to part-time work (17%), and eight had not returned to work (35%). Of the six patients working in part-time or casual positions at diagnosis, four returned to work and two did not. Of the ten people that did not return to work, the most common reason for not returning to work was adverse side effects from treatment (90%). Nineteen patients (33%) reported that a member of their treating team had asked if they had any financial concerns. Most people reported being very comfortable speaking to a member of their medical team about financial difficulties, with a median score of 10 on a 10-point Likert scale. 

**Figure 2:**
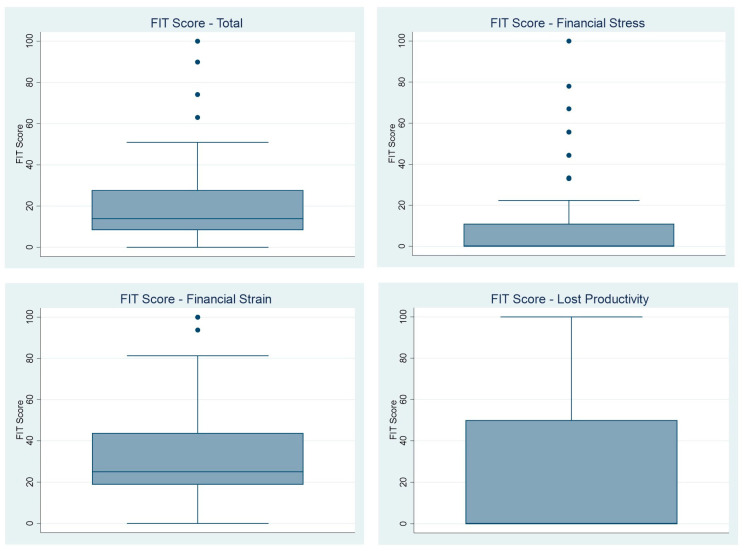
FIT Scores. Note: Higher scores indicate that higher financial toxicity.

The overall median FIT score was 13.9 (19.5), with a median score of 0 (11) for financial stress, 25 (25) for financial strain, and 0 (50) for lost productivity (Table 2, Figure 2). Patients who were not married (included those who were divorced, single, or widowed) had a higher financial toxicity score (23.1 vs. 11.1, *p* = 0.01). Additionally, participants with private health insurance had lower rates of financial toxicity (8.3 vs. 17.6, *p* = 0.01). Participants with lower levels of education had higher rates of financial toxicity (19.3 vs. 11.1, *p* = 0.06). No differences in FIT scores were found for the other sociodemographic or clinical factors. As demonstrated in Table 3, the 14 patients with high levels of FT (highest 25% of FIT scores), had a median FIT score of 44.4, compared with a median of 11.1 in the low FT group. Patients in the high FT group were younger (median age 57 vs. 65, *p* = 0.04) and were more likely to be not married (71% vs. 33%, *p* = 0.011) compared to those in the low FT group. The patients with high FT had worse quality of life across all FACT domains apart from the head and neck cancer subscale scores.

There were wide variations in out-of-pocket expenses in the first 12 months after diagnosis between participants (Table 4). A total of 15 (27%) patients reported zero out-of-pocket expenses. Of the 41 (73%) patients who reported out-of-pocket expenses, the median was AUD 1796 (IQR—AUD 2700), with a maximum expense of AUD 25,050. The most common out-of-pocket median expenses for patients were medications (AUD 400), dietary supplements (AUD 600), travel (AUD 525), and accommodation (AUD 520). Table 5 shows that participants living in rural locations (≥100 km from Townsville Hospital) had higher out-of-pocket expenses (AUD 2655 vs. AUD 730, *p* = 0.01). Participants with higher levels of education also had higher out-of-pocket expenses (AUD 2680 vs. AUD 1167, *p* = 0.03), as well as those with an annual income greater than AUD 45,000 (AUD 2740 vs. AUD 1134, *p* = 0.01). Patients working at diagnosis had higher out-of-pocket expenses than those not working (AUD 2655 vs. AUD 730, *p* = 0.01). Recipients of chemoradiotherapy had higher out-of-pocket expenses than those who received radiotherapy alone or surgery and radiotherapy (AUD 1850 vs. AUD 600, *p* = 0.04).

## 4. Discussion

Overall, there were low levels of financial toxicity across the study cohort, although a proportion of patients reported significant financial concerns. Patients with higher financial toxicity reported poorer HRQoL. Out-of-pocket costs within the first 12 months of diagnosis varied widely between patients. While some reported no costs, out-of-pocket expenses were incurred at a median of AUD 1796 by 41 patients. Patients from a lower socioeconomic background (those not working and with a lower education level) had reduced out-of-pocket expenses, which was likely due to government concessions and bulk-billing of healthcare services. However, patients from rural locations had increased out-of-pocket expenses, highlighting this as a group of people who may require additional financial support. Medications, dietary supplements, travel, and dental were the most common causes of out-of-pocket expenses.

Currently, one other study has reported FIT scores for patients with head and neck cancer, and this was the validation study by Hueniken et al. [13]. The authors of this validation study found a median FIT score of 11.1, which is similar to the score reported in the current study. Other studies have assessed the financial toxicity of patients with head and neck cancer using the Comprehensive Score For Financial Toxicity (COST), although most of these studies were conducted in the USA [9]. Our study found that not being married and no private health insurance were factors associated with an increased FIT score, which is consistent with the literature [9,11,19]. There was an association between FT and HRQoL in this study, with patients who demonstrated high levels of financial toxicity found to have inferior HRQoL across most domains. A previous systematic review has also highlighted the impacts of financial toxicity on quality of life, in particular mental wellbeing [6]. Methods in which to improve rates of financial toxicity are therefore required, even in publicly funded healthcare systems such as Australia. A study in the USA demonstrated that a dedicated financial counsellor resulted in reduced financial difficulty scores in patients with head and neck cancer [20]. Other studies have explored the role of using general practitioners to address financial toxicity amongst patients with cancer [21].

The median out-of-pocket expense per participant was AUD 1796, which is similar with other studies in the literature. A prospective longitudinal study performed in Canada found that the median out-of-pocket cost for patients undergoing chemoradiation was CAD 1455, radiation therapy alone was CAD 630, and surgery with adjuvant radiotherapy was CAD 1626 [22]. Our study also demonstrated that patients receiving chemoradiation had higher costs compared to RT alone or surgery and RT.

Medications, dietary supplements, travel, and dental were the most common items that incurred out-of-pocket expenses for participants in our study. Within the current funding arrangements in Australia, medications and travel are both subsidised but these can be variable, and patients are often left with an out-of-pocket cost as seen in our study. Despite the subsidy, travel and accommodation costs can become substantial. The Patient Travel Subsidy Scheme in Queensland covers a proportion of travel and accommodation costs, but those living far from the health facility had substantially increased out-of-pocket expenses. This is concerning given that RT for patients with HNC is generally 6 to 7 weeks in duration, meaning that accommodation costs can be significant. Given that RT for HNC is offered only in major centres in Queensland, it is common for patients to need to travel for treatment, as evident by the fact that 55% of patients in this study lived more than 100 km from their treatment centre. More financial support is needed to ensure these patients do not continue to be disproportionately affected. Dietary supplements are currently not subsidised and were the highest expense for participants. Most patients require dietary supplements at some stage during RT, with many patients being dependent upon these during treatment and in the weeks after. Government funding for dental services is severely lacking in Australia leading to out-of-pocket costs as shown in this study. Patients with HNC treated with RT are at risk of long-term dental complications including osteoradionecrosis which has significant morbidity for affected individuals. Although not explored in this study, it is possible that high out-of-pocket costs for dental services may result in patients forgoing regular appointments, placing them at higher risk of future complications. More urgent attention is needed in Australia to ensure that patients treated with RT can access regular government-subsidised dental services as part of their survivorship care.

Specialist appointments or fees only accounted for a low percentage of out-of-pocket expenses, and this is likely because this study was conducted in a public hospital where most of these expenses are covered. The higher out-of-pocket costs incurred by patients who were higher educated, had a higher annual household income, and were working at the time of diagnosis could be because this cohort opted to access more services (such as allied health and/or private specialists) that incur out-of-pocket expenses or because they did not receive concessions or bulk-billing of services. Patients receiving the aged pension or those who were unemployed may also have had lower out-of-pocket expenses due to increased government financial assistance, subsidies, and bulk-billing health providers.

Only one third of participants reported being asked by their treating team if they had any financial concerns. However, patients reported being very comfortable discussing financial difficulties with their medical team. This shows there needs to be more initiative taken by health professionals to discuss financial concerns and support options with patients. A recent Australian survey of health professionals investigating clinician perspectives towards addressing cancer-related financial toxicity highlighted that 88% of participants believed discussing financial concerns was important. Common barriers to discussing financial issues were lack of time by health professionals and insufficient resources or support services to refer their patients to [23]. More research is required to determine which health professionals are best placed to have financial discussions with patients and how this can be integrated into routine clinical practice.

This study was conducted in a single, publicly funded hospital with a small sample size. Given the inherent limitations of cross-sectional analysis, it was not possible to assess whether financial toxicity altered over time, although it is known that healthcare costs are highest in the first 12 months since diagnosis [24]. Another limitation was recall bias, where participants needed to remember their out-of-pocket expenses that were incurred 12 to 36 months previously. Due to these reasons, it is very likely that the out-of-pocket expenses are higher than reported and these figures are an under-representment. While our response rate was high, we did not capture all eligible patients and therefore our results may be subject to some selection bias. Finally, our study was not powered to detect statistically significant findings across high and low financial toxicity groups, despite finding significant differences, and these findings should be viewed cautiously. These drawbacks should be balanced against the study strengths which include a high response rate among patients with a less common type of cancer, providing detailed cost data, and survey responses on validated tools.

Future research should consider prospectively measuring financial toxicity and out-of-pocket costs in patients with HNC to consider how these change over the course of treatment and follow-up period. Studies focusing on specific subsites of HNC are required to determine whether there are differences in financial concerns between these groups. Research investigating interventions to reduce financial toxicity specific to patients with HNC is also needed. Clinical teams providing care to patients with head and neck cancer should consider screening for financial concerns at the initial assessment and again during follow-up as part of routine clinical care [23]. All clinicians should consider asking patients with HNC about financial concerns and be aware of what financial support is available in their region, as well as being advocates to guide policy changes. Government funding for dental services for all head and neck cancer patients is needed, as well as improved travel and accommodation subsidies to ensure regional and rural patients are not disadvantaged.

## 5. Conclusions

Financial toxicity is a significant issue for a proportion of patients with head and neck cancer, which was associated with a reduced HRQoL. Patients who were not married and those without private health insurance experienced higher financial toxicity. Dietary supplements, medications, travel, and dental were common out-of-pocket costs, with regional and rural patients having increased expenses. Interventions are required to reduce the burden of financial toxicity for patients with HNC.

## Figures and Tables

**Table 1:** Summary of Patient and Clinical Characteristics.

Patient Characteristics (*n* = 57)	*n* (%)	Patient Characteristics (*n* = 57)	*n* (%)
Age (at survey completion) Mean + SD	63.8 + 9.2	Time from treatment completion to survey (years) Mean + SD	1.9 + 0.63
Sex		Private health insurance *	19 (34%)
Male	43 (75%)	Yes	37 (66%)
Female	14 (25%)	No	19 (34%)
Ethnicity		Total annual household income (diagnosis)	
Aboriginal	1 (2%)	0 to AUD 18,200	9 (16%)
Torres Strait Islander	0 (0%)	AUD 18,201 to AUD 45,000	21 (37%)
Both Aboriginal and Torres Strait Islander	1 (2%)	AUD 45,001 to AUD 120,000	17 (30%)
Neither	55 (96%)	>AUD 120,000	10 (18%)
Distance to Townsville Hospital	23 (40%)	Employment status (at diagnosis)	
<50 km	3 (5%)	Full-time work	23 (40%)
50–99 km	3 (5%)	Part-time work	2 (4%)
100–199 km	7 (12%)	Casual work	4 (7%)
200–299 km	13 (23%)	Retired	24 (42%)
300–399 km	8 (14%)	Unemployed	3 (5%)
≥400 km	23 (40%)	Volunteer/unpaid work	1 (2%)
Charlson Comorbidity Index (CCI) Mean + SD	2.5 + 1.6	Sole income earner *	
		Yes	27 (48%)
		No	29 (52%)
ECOG		Self-employed	
0 1	26 (46%) 31 (54%)	Yes No N/A (not working)	10 (18%) 35 (61%) 12 (21%)
Smoking status		Marital status	
		Married/de facto	33 (58%)
Current	19 (33%)	Divorced or separated	15 (26%)
Former	24 (42%)	Single or never married	6 (11%)
Never	14 (25%)	Widowed	3 (5%)
Level of education		Occupation (ISCO-08 classification)	
Primary School	5 (9%)	Retired or Unemployed	15 (26%)
		Managers	6 (11%)
Secondary School (Grade 10)	20 (35%)	Professionals	3 (5%)
		Technicians	4 (7%)
Secondary School (Grade 12)	6 (11%)	Service and Sales Workers	7 (12%)
		Agricultural, Forestry, and Fishery	2 (4%)
Technical College, Diploma, or Certificate	21 (37%)	Craft Workers	6 (11%)
		Plant and Machine Operators	10 (18%)
University Degree	5 (9%)	Elementary Occupations	4 (7%)
Living arrangements			
Live with partner	25 (44%)		
Live alone	21 (37%)		
Live with partner and children	6 (11%)		
Live with children	2 (4%)		
Assisted living	1 (2%)		
Other	2 (4%)		
**Clinical Characteristics**			
Tumour location			
Oral cavity	7 (12%)		
Nasopharynx	2 (4%)	Treatment	
Oropharynx–p16-positive	30 (53%)	Chemo RT	35 (61%)
Oropharynx–p16-negative	7 (12%)	RT alone	11 (19%)
Hypopharynx	2 (4%)	Surgery + RT	7 (12%)
Larynx	8 (14%)	Surgery + chemo RT	4 (7%)
Unknown	1 (2%)		
Tumour stage (AJCC 8th edition)			
I	16 (28%)		
II	12 (21%)		
III	16 (28%)		
IV	1 (2%)		
IVA	11 (19%)		
IVB	1 (2%)		
**Financial Support**			
Has a member of your treating team asked if you have any financial concerns?			
Yes			19 (33%)
No			38 (67%)
How comfortable are you in discussing financial difficulties with your medical team?			
Median (SD)			10 (3) *
Financial support during RT		Financial support 12 months after treatment	
Worked during treatment	3 (5%)	Returned to work	18 (32%)
Sick leave	7 (12%)	Sick leave	1 (2%)
Superannuation	6 (11%)	Superannuation	4 (7%)
Income protection	4 (7%)	Income protection	4 (7%)
JobSeeker **	7 (12%)	JobSeeker **	5 (9%)
Age pension	21 (37%)	Pension	24 (42%)
Disability pension	4 (7%)	Other	8 (14%)
Other	14 (25%)		
Employment status 12 months post cancer treatment		Income 12 months post cancer treatment	
Full-time work	11 (19%)	Same income	22 (39%)
Part-time work	8 (14%)	Lower income	13 (23%)
Did not return to work	10 (18%)	Higher income	2 (4%)
Not working previously	28 (49%)	N/A—not working before diagnosis	20 (35%)
Reasons for not returning to work (*n* = 10) ^			
Side effects from treatment	9 (90%)		
Work-related issues	1 (10%)		
Other	1 (10%)		

* Missing data for 1 participant. ** JobSeeker is an Australian-government-funded payment for people who are unemployed or are sick or injured for a period of time. ^ Multiple options allowed.

**Table 2:** Financial Toxicity (FIT) Scores by sociodemographic and clinical variables, median (IQR).

FIT Scores *		
Total Score—13.9 (19.5)		
Financial Stress—0 (11)		
Financial Strain—25 (25)		
Lost Productivity—0 (50)		
Factors Associated with Financial Toxicity		
Variable	FIT Total Scores *	*p* Value
Age		0.99
≤60	13.9 (38.1)	
>60	13.9 (13.9)	
Sex		0.14
Male	12 (19.5)	
Female	18.4 (19.5)	
Distance to Townsville Hospital		0.47
0–99 km	17.1 (13.9)	
>100 km	12 (22.3)	
Smoking Status		
Current	19.4 (35.3)	0.23
Former/Never	13.9 (11.1)	
Level of Education		0.06
Primary/High School	19.3 (25.1)	
TAFE/University	11.1 (12)	
Marital Status		**0.01**
Married	11.1 (11)	
Not Married	23.1 (34.2)	
Private Health Insurance		**0.01**
Yes	8.3 (13.8)	
No	17.6 (22.3)	
Annual Household Income		0.10
≤AUD 45,000	17.1 (16.7)	
>AUD 45,000	11.1(24.9)	
Sole Income Earner		0.12
Yes	17.6 (36)	
No	12 (11.1)	
Employment Status at Diagnosis		0.37
Working	17.6 (31.5)	
Not Working	12 (11.1)	
Self-Employed		0.80
Yes	15.3 (19.5)	
No or N/A	13.9 (22.3)	
Tumour Location		0.90
Oropharyngeal	15.7 (19.5)	
Other	13.9 (18.1)	
Tumour Stage		0.85
Early	18.4 (22.3)	
Advanced	13.9 (13.9)	
Treatment Received		0.60
ChemoRT ± Surgery	13.9 (22.2)	
RT alone or Surgery + RT	12.5 (25.1)	

* Scores reported as median (IQR). A higher score indicates a higher level of financial toxicity.

**Table 3:** Quality of Life and Financial Toxicity.

Quality of Life—FACT H&N					
	Overall Cohort Median (IQR)	High FT (*n* = 14) Median (IQR)	Low FT (*n* = 42) Median (IQR)	*p* Value	Spearman’s Rho
Physical Wellbeing	23 (8)	16 (10)	24.5 (6)	**<0.001**	−0.52
Social/Family Wellbeing	22 (9.5)	15.6 (11)	24 (8.2)	**<0.001**	−0.55
Emotional Wellbeing	20 (6.5)	16 (7)	20 (6)	**0.004**	−0.40
Functional Wellbeing	18.5 (9)	13.5 (8)	21 (9)	**0.002**	−0.45
Head and Neck Cancer Subscale	25 (11)	22 (16)	25 (10)	0.13	−0.29
Total Score	105.5 (33.4)	76.5 (43.2)	114.5 (29)	**<0.001**	−0.55

Note—High FT taken as the top 25% of scores on the FIT score (*n* = 14). Median FIT scores (High FT = 44.4, Low FT = 11.1).

**Table 4:** Out-of-Pocket Expenses.

Out-of-Pocket Expenses	Number of Participants with Out-of-Pocket Expenses (%) *n* = 56	Total Expense (12-Month Period) *
Dental	16 (29%)	388 (825)
Allied Health	5 (9%)	500 (3284)
Mental Health	3 (5%)	300 (440)
GP	15 (27%)	250 (380)
Specialist	7 (13%)	600 (1100)
Accommodation	10 (18%)	520 (300)
Travel	20 (36%)	525 (825)
Home Services	6 (11%)	716 (700)
Dietary Supplements	23 (41%)	600 (950)
Investigations	8 (14%)	575 (1190)
Medications	23 (41%)	400 (600)
Total	41 (73%)	1796 (2700)
		Minimum—18
		Maximum 25,050

* Median (IQR), excludes patients who had no out-of-pocket expenses for that particular expense, reported in AUD.

**Table 5:** Factors Associated with Out-of-Pocket Expenses.

Variable	Out-of-Pocket Expense *	*p* Value
Age		0.20
≤60	2000 (2500)	
>60	1400 (3078)	
Sex		0.53
Male	1570 (2500)	
Female	1798 (3585.5)	
Distance to Townsville Hospital		**0.01**
0–99 km	730 (1300)	
≥100 km	2655 (2450)	
Smoking Status		0.25
Current	1400 (2900)	
Former/Never	1825 (2606)	
Level of Education		**0.03**
Primary/High School	1167 (1380)	
TAFE/University	2680 (2230)	
Marital Status		0.44
Married	1500 (2750)	
Not Married	1865 (2233)	
Private Health Insurance		0.12
Yes	2680 (2400)	
No	1400 (2700)	
Annual Household Income		**0.01**
≤AUD 45,000	1134 (1878)	
>AUD 45,000	2740 (2492)	
Sole Income Earner		0.42
Yes	1850 (2666)	
No	1300 (2250)	
Employment Status at Diagnosis		**0.01**
Working	2655 (2684)	
Not Working	730 (1890)	
Self-Employed		0.33
Yes	3185 (2858)	
No or N/A	1632 (2250)	
Tumour Location		0.69
Oropharyngeal	1800 (3010)	
Other	1714 (2650)	
Tumour Stage		0.23
Early	1500 (2300)	
Advanced	2000 (3354)	
Treatment Received		**0.04**
ChemoRT ± Surgery	1850 (2600)	
RT alone or Surgery + RT	600 (2035)	

* Median (IQR), reported in AUD.

## Data Availability

The data that support the findings of this study are available from the corresponding author upon reasonable request.

## Supplementary Materials

The following supporting information can be downloaded at: https://www.mdpi.com/article/10.3390/curroncol30050371/s1, Supplementary Questionnaire S1: Toxicity and Out of Pocket Expenses in Patients with Head & Neck Cancer.
