# Supplementary material for: Financial Toxicity and Out-of-Pocket Costs for Patients with Head and Neck Cancer
Source: Curr Oncol. 2023 May 10;30(5):4922–35. doi: 10.3390/curroncol30050371 (PMC10217128; doi:10.3390/curroncol30050371)
Supplement: Supplementary file 1 [file curroncol-30-00371-s001.zip › curroncol-2330210-supplementary.pdf]

Patient No. _________

Financial Toxicity and Out of Pocket Expenses in Patients with Head & Neck Cancer

Patient Questionnaire

Date Completed: ___________

Financial toxicity is recognised as the financial burden that results from a cancer diagnosis and treatment. The following questions relate to your experiences regarding financial matters before and during your treatment.

Section 1: General Information

Time of Diagnosis

1. What is your highest level of education attained (please tick 1 option below)?
   - ☐ Primary School
   - ☐ Secondary School (Grade 10)
   - ☐ Secondary School (Grade 12)
   - ☐ Apprenticeship, TAFE, diploma or certificate training
   - ☐ University Degree
2. What is your marital status?
   - ☐ Married/de facto
   - ☐ Divorced or separated
   - ☐ Single or never married
   - ☐ Widowed
3. What are your living arrangements (please tick all that apply)?
   - ☐ Live with partner
   - ☐ Live alone
   - ☐ Live with partner and children
   - ☐ Live with children
   - ☐ Assisted living
   - ☐ Other (please specify ______________)
4. Do you currently smoke (have you smoked a single cigarette in the last 30 days)?
   - ☐ Yes
   - ☐ No
5. At the time of your head and neck cancer diagnosis did you have private health insurance (please tick 1 option below)?

   Yes No
6. Do you have a Health Care card?
   - ☐ Yes, I have had one since before my head and neck cancer diagnosis
   - ☐ Yes, I have one now but didn't have one at the time of my head and neck cancer diagnosis
   - ☐ No
7. At the time of your head and neck cancer diagnosis what was your total annual household income (please include you and your partner's income) before tax?
   - ☐ 0 - $18,200

- ☐ $18,201 to $45,000
- ☐ $45,001 to $120,000
- ☐ $120,001 to 180,000
- ☐ $180,001 and over

8. Are you the sole income earner in your household?
   - ☐ Yes
   - ☐ No
9. At the time of your head and neck cancer diagnosis what was your employment status?
   - ☐ Full-time work
   - ☐ Part-time work (if Yes, please state the number of hours per week _______)
   - ☐ Casual work
   - ☐ Retired
   - ☐ Unemployed
   - ☐ Volunteer/unpaid work
10. What was your occupation at the time of your diagnosis?

    ____

11. Were you self-employed?
    - ☐ Yes
    - ☐ No
    - ☐ Not applicable, I was not working

Please respond to the following questions regarding how your income and work hours were affected during and after treatment.

<u>During Treatment</u>

12. Were you working during treatment (for patients receiving radiotherapy)
    - ☐ Yes, I worked the same number of hours
    - ☐ Yes, I worked a reduced number of hours
    - ☐ No, I stopped work during treatment
    - ☐ Not applicable, I was not working before treatment
13. How did you financially support yourself during treatment (please tick all that apply)
    - ☐ I worked during treatment (even if you worked for part of your treatment)
    - ☐ Sick leave
    - ☐ Superannuation
    - ☐ Income protection
    - ☐ JobSeeker
    - ☐ Age pension
    - ☐ Disability pension
    - ☐ Other financial sources – (please specify ________________)

<u>After Treatment</u>

14. How did you financially support yourself in the 12 months after your treatment (please tick all that apply)
    - ☐ I returned to work
        - How many months after treatment did you return to some form of work (e.g. part time) _______
        - How many months after treatment did you return to full time work ______
    - ☐ Sick leave
    - ☐ Superannuation
    - ☐ Income protection
    - ☐ Newstart
    - ☐ Pension
    - ☐ Other financial sources – (please specify _________________)
15. What was your employment status at 1 year after your cancer treatment had finished?
    - ☐ I have returned to work full time
    - ☐ I have returned to work part time (how many hours per week_____)
    - ☐ I did not return to work
    - ☐ I was not working previously
16. How was your income affected at 1 year after your cancer treatment had finished?
    - ☐ Same income
    - ☐ Lower income
    - ☐ Higher income
    - ☐ Not applicable – I was not working before my diagnosis
17. If you were working at the time of your diagnosis and did not return to work after treatment what were the reasons?
    - ☐ Side effects from treatment - please specify further:

      ____
    - ☐ Work related issues (e.g. example unsupportive environment. Please write details below:

      ____
    - ☐ Other (please explain below)

      ____
18. At any time before or during your treatment has a member of your treatment team asked if you have any financial concerns?
    - ☐ Yes
    - ☐ No
19. How comfortable are you in discussing financial difficulties with your medical treatment team? Please rate this on a scale from 1 (not very comfortable) to 10 (very comfortable).

    1 2 3 4 5 6 7 8 9 10

Section 2: Financial Index of Toxicity Questionnaire

The following questions relate to what has occurred in the past 1 year

1. During the past year, were you satisfied with your family's financial situation?	☐ Very satisfied<br>☐ Somewhat satisfied<br>☐ Neutral<br>☐ Somewhat dissatisfied<br>☐ Very dissatisfied
2. During the past year, were you worried about your family's financial situation?	☐ Neutral<br>☐ Somewhat worried<br>☐ Very worried
3. How did you perceive your family's financial situation compared to others of the same age?	☐ Far worse<br>☐ Somewhat worse<br>☐ The same<br>☐ Somewhat better<br>☐ Far better
4. During the past year, were you able to pay for your daily food expenses?	☐ All of the time<br>☐ Most of the time<br>☐ Some of the time<br>☐ None of the time
5. During the past year, were you able to pay for your daily housing expenses?	☐ All of the time<br>☐ Most of the time<br>☐ Some of the time<br>☐ None of the time
6. During the past year, were you able to pay for your medications related to your treatment?	☐ All of the time<br>☐ Most of the time<br>☐ Some of the time<br>☐ None of the time
7. During the past year, did you have to borrow money to help with treatment related expenses from family/friends/financial institutions?	☐ Yes<br>☐ No
8. Have you had to permanently quit your previous occupation because of your cancer diagnosis and/or treatment?	☐ Yes<br>☐ No
9. Did any of your family or friends have to permanently quit their job to help with your care needs because of your cancer diagnosis or treatment?	☐ Yes<br>☐ No

Section 3 – FACT-H&N (Version 4)

Below is a list of statements that other people with your illness have said are important. **Please circle or mark 1 number per line to indicate your response as it applies to the past 7 days**

Physical Well-Being	Not at all	A little bit	Some-what	Quite a bit	Very much
1. I have a lack of energy	0	1	2	3	4
2. I have nausea	0	1	2	3	4
3. Because of my physical condition, I have trouble meeting the needs of my family	0	1	2	3	4
4. I have pain	0	1	2	3	4
5. I am bothered by side effects of treatment	0	1	2	3	4
6. I feel ill	0	1	2	3	4
7. I am forced to spend time in bed	0	1	2	3	4

Social/Family Well-Being	Not at all	A little bit	Some-what	Quite a bit	Very much
1. I feel close to my friends	0	1	2	3	4
2. I get emotional support from my family	0	1	2	3	4
3. I get support from my friends	0	1	2	3	4
4. My family has accepted my illness	0	1	2	3	4
5. I am satisfied with family communication about my illness	0	1	2	3	4
6. I feel close to my partner (or the person who is my main support)	0	1	2	3	4
Regardless of your current level of sexual activity, please answer the following question. If you prefer not to answer it, please mark this box and go to the next section.					
7. I am satisfied with my sex life	0	1	2	3	4

Emotional Well-Being	Not at all	A little bit	Some-what	Quite a bit	Very much
1. I feel sad	0	1	2	3	4
2. I am satisfied with how I am coping with my illness	0	1	2	3	4
3. I am losing hope in the fight against my illness	0	1	2	3	4
4. I feel nervous	0	1	2	3	4
5. I worry about dying	0	1	2	3	4
6. I worry that my condition will get worse	0	1	2	3	4

Patient No. _________

Functional Well-Being	Not at all	A little bit	Some-what	Quite a bit	Very much
1. I am able to work (include work at home)	0	1	2	3	4
2. My work (include work at home) is fulfilling	0	1	2	3	4
3. I am able to enjoy life	0	1	2	3	4
4. I have accepted my illness	0	1	2	3	4
5. I am sleeping well	0	1	2	3	4
6. I am enjoying the things I usually do for fun	0	1	2	3	4
7. I am content with the quality of my life right now	0	1	2	3	4

Additional Concerns	Not at all	A little bit	Some-what	Quite a bit	Very much
1. I am able to eat the foods that I like	0	1	2	3	4
2. My mouth is dry	0	1	2	3	4
3. I have trouble breathing	0	1	2	3	4
4. My voice has its usual quality and strength	0	1	2	3	4
5. I am able to eat as much food as I want	0	1	2	3	4
6. I am unhappy with how my face and neck look	0	1	2	3	4
7. I can swallow naturally and easily	0	1	2	3	4
8. I smoke cigarettes or other tobacco products	0	1	2	3	4
9. I drink alcohol (e.g. beer, wine, etc.)	0	1	2	3	4
10. I am able to communicate with others	0	1	2	3	4
11. I can eat solid foods	0	1	2	3	4
12. I have pain in my mouth, throat or neck	0	1	2	3	4

English (Universal)
Copyright 1987, 1997

Section 4: Out-Of-Pocket Expenses

Since your head and neck cancer diagnosis indicate the number of times you have accessed the below service and the out of pocket costs that this has incurred. These costs must be those that were not covered by Medicare, your private health insurance, or any other source. The costs must be related to your head and neck cancer diagnosis or treatment and be within the **first 12 months of diagnosis (including the period of time you were having radiotherapy)**. Please estimate the total out of pocket expense for the entire 12 month period (for example if you required accommodation during your radiation treatment (7 weeks) and then for 1 follow up visit and were out of pocket $100/week during treatment and $100 for 1 night accommodation then this would be $800. If you have had no out of pocket expenses for a service, then please write 0. If you have had other out of pocket expenses, please write these in the “Other” category and specify what the service was.

Type of Service	Number of Visits/ Times Accessing Service	Out of Pocket Expense (in $)	Have you not accessed this service because of cost?
Dental			
Allied Health (Physiotherapy, Occupational Therapy)			
Mental Health (counselling, psychologist, psychiatrist)			
Medical – General practitioner			
Medical – specialist appointments			
Accommodation			
Travel (e.g. cost of fuel, flights)			
Home services (e.g. assistance cleaning)			
Dietary supplements			
Investigations – e.g. blood tests, scans			
Medications (e.g. pain medications)			
Other			
